# A systematic review protocol for measuring comorbidity in inpatient rehabilitation for non-traumatic brain injury

**DOI:** 10.1186/2046-4053-4-14

**Published:** 2015-01-29

**Authors:** Wayne Khuu, Vincy Chan, Angela Colantonio

**Affiliations:** Toronto Rehabilitation Institute, University Health Network, 550 University Avenue, Toronto, Ontario M5G 2A2 Canada; Dalla Lana School of Public Health, University of Toronto, 155 College Street, Toronto, Ontario M5T 3 M7 Canada; Rehabilitation Sciences Institute, University of Toronto, 500 University Avenue, Toronto, Ontario M5G 1 V7 Canada

**Keywords:** Brain injury, Rehabilitation, Health services, Comorbidity, Risk adjustment, Epidemiology, Systematic review

## Abstract

**Background:**

Comorbidity can affect health-care utilization and outcomes, and the results and interpretation of risk adjustment studies that attempt to predict rehabilitation utilization and outcomes are influenced by the choice of comorbidity measurement. Although the identification of an appropriate measurement has been conducted in some populations and outcomes, this information is currently lacking for the non-traumatic brain injury (nTBI) population in inpatient rehabilitation settings. As such, this is a systematic review protocol to survey the methods used to measure comorbidities in the rehabilitation setting for patients with nTBI.

**Methods/design:**

MEDLINE, MEDLINE In-Process, Embase, The Cochrane Database of Systematic Reviews, PsycINFO, and Health and Psychosocial Instruments will be systematically searched using the concepts ‘nTBI,’ ‘comorbidity,’ and ‘rehabilitation.’ Grey matters and the reference list of eligible articles will also be searched. Study selection will be performed independently by two reviewers based on predetermined eligibility criteria through two rounds of screening using, first, the title and abstract, followed by full-text. Extracted information will include study purpose, design, and setting; data source and type; outcomes variables; statistical methods; comorbidity measurement method, rationale, justification, or validation; and results involving comorbidity. The data will be tabulated and narratively synthesized. Meta-analyses will be performed if appropriate.

**Systematic review registration:**

This protocol has not been registered with PROSPERO.

**Discussion:**

This protocol provides a systematic method for surveying current practice as well as monitoring the progress on comorbidity measurement methodology and effects of comorbidity on rehabilitation outcomes for patients with nTBI. The selection of an appropriate comorbidity measurement method has implications for the interpretation of both descriptive and risk adjustment studies, and thus, the validity of evidence used to inform planning and delivery of services.

**Electronic supplementary material:**

The online version of this article (doi:10.1186/2046-4053-4-14) contains supplementary material, which is available to authorized users.

## Background

Non-traumatic brain injury (nTBI) is defined as acquired brain damage occurring after birth due to cancer, anoxia, infection or inflammation, toxicity, and vascular insults [1]. Approximately 10% of patients with nTBI are discharged to inpatient rehabilitation after hospitalization [2], and the direct medical costs of rehabilitation account for approximately one third of health-care costs in the first year after injury [3]. As such, it is important to identify predictors of rehabilitation outcomes to inform the health-care needs of this population [4, 5].

Comorbidity is often considered in studies that assess rehabilitation outcomes as they can affect health service utilization [6] and functional outcomes [7]. Examples of common indexes used to measure comorbidities include the Charlson Comorbidity Index (CCI) [8] and Elixhauser Index [9] and more complex case-mix risk adjustment models that include comorbidity information such as the Centers for Medicare and Medicaid Service (CMS) Comorbidity Tiers [10, 11]. These comorbidity measurement methods were developed for specific outcomes, namely cost or mortality, and in specific patient populations and settings. For example, the original CCI includes 22 conditions that were found to be significantly associated with 1-year mortality in a sample of 607 patients admitted to a single medical center in New York in 1984. It was later validated in a sample of 685 female breast cancer patients [8] and recently updated with reassigned weights in a more contemporary setting [12]. The CMS Comorbidity Tiers was developed across all inpatient rehabilitation patients according to cost of treatment using data from 1998 [10] and later updated using data from 2003, which resulted in a different set of comorbidities for tier definition [11].

As an emerging field of study, various comorbidity measures have been compared against each other to assess their predictive power for certain populations and outcomes. For example, among the CCI, Elixhauser Index, and Johns Hopkins Aggregated Diagnosis Groups (ADGs), the Johns Hopkins ADGs showed marginally higher discrimination for predicting 1 year all-cause mortality in patients with chronic obstructive pulmonary disorder [13]. In a sample of medical, procedural, and psychiatric inpatients, the Multipurpose Australian Comorbidity Scoring system performed better than the CCI in predicting mortality, readmission, and length of stay [14]. Within elderly individuals, the CCI, Cumulative Illness Rating Scale-Geriatrics, Index of Coexistent Diseases, Kaplan, Geriatric Index of Comorbidity, and Chronic Disease Score showed differential predictive power for mortality, institutionalization, and readmission [15]. Furthermore, systematic reviews that have assessed the validity of comorbidity indexes in specific populations, such as patients with acute myocardial infarction, heart failure, and stroke [16], and specific outcomes, such as mortality [17], demonstrate that certain comorbidity measurements are more valid for some populations than others.

To date, no such analysis has been published for rehabilitation outcomes after a nTBI. Since an absence of evidence is not evidence of absence, it should not be assumed that a given comorbidity measurement method is more or less appropriate for inpatient rehabilitation outcomes such as length of stay and functional outcome following a nTBI, especially as this population differs in various characteristics from those that are not referred to an inpatient rehabilitation setting [2]. Indeed, other authors have acknowledged that ‘future research is needed to examine the impact of comorbidities and complications on outcomes of other [Inpatient Rehabilitation Facilities] populations to better understand the implications for current and future health-care policy’ [18].

The breadth of patients included in the definition of nTBI may preclude the discovery of important differences in the appropriateness of specific comorbidity measures between specific subgroups of patients with nTBI. It is expected that certain indexes may be less appropriate for certain patient groups. For example, since tumors are scored in the CCI, it may be less appropriate for patients with brain tumors because their comorbidity score will be inflated. Therefore, it is important to investigate subtypes of nTBI separately, as well as patients with nTBI as a whole, which reflects clinical practice where patients with nTBI are treated in the same rehabilitation unit. The inclusion of all subtypes of nTBI in this protocol will allow for investigations of comorbidity measurement methods within and between subtypes of nTBI.

The primary purpose of this protocol is to systematically review the literature to identify current comorbidity measurements used for the nTBI population in inpatient rehabilitation. The secondary objective is to assess the face and predictive validity of these comorbidity measurements for various rehabilitation outcomes, including functional outcome and length of stay, specifically for this population. The final and tertiary objective is to catalogue the profile of comorbidities in patients with nTBI in inpatient rehabilitation settings using the identified studies. This information can be used to inform efforts to develop predictive multivariable models of rehabilitation outcomes for health service research, in which the selection of the most valid comorbidity measurement specific to the nTBI population in the rehabilitation setting can improve the clinical relevance and predictive power of the risk adjustment models for rehabilitation outcomes of these patients.

## Methods/design

### PROSPERO

This protocol has not been registered with PROSPERO.

### Search strategy

The search strategy was developed with assistance from a medical librarian.

The following databases will be searched for relevant articles:

1. MEDLINE (1946 to present) and MEDLINE In-Process (present)
2. Embase (1946 to present)
3. Cochrane Database of Systematic Reviews (2005 to present)
4. PsycINFO (1806 to present)
5. HAPI (1985 to present)

Grey literature will also be searched using Grey Matters: a practical search tool for evidence based medicine [19]. Resources from known relevant national rehabilitation research groups (e.g., The United States Centers for Medicare and Medicaid Services and Canadian Institute for Health Information) will be used to survey available information from health service providers. Finally, the reference list of included articles will be searched.

Additional file 1 provides the search strategy for each database. The search strategy combines the concepts ‘nTBI,’ ‘comorbidity,’ and ‘rehabilitation.’ Broad terms to define nTBI are used to increase sensitivity, and more narrow terms are used to define specific nTBI conditions. The terms for comorbidity are based on Buck et al. [16] and supplemented with risk adjustment concepts related to health service research, such as ‘diagnosis related groups,’ ‘case-mix,’ and ‘resource intensity weighting,’ as they may incorporate comorbidity information.

All database results will be limited to English language. A limit to human only studies will be applied in MEDLINE, and limits to exclude conference material (abstracts, paper, proceeding, and reviews), editorials, letters, and notes will be applied in Embase.

### Study selection

Two reviewers will independently assess all titles and abstracts for inclusion using predetermined eligibility criteria. Two iterations of screening will be performed. First, record titles (and abstracts if the title is ambiguous) will be used to select articles that study a nTBI population in inpatient rehabilitation. Second, a full-text screen will be used to identify relevant articles for inclusion in the systematic review. Articles that are included in the systematic review must (1) examine comorbidities in the nTBI population in inpatient rehabilitation and (2) state the method in which these comorbidities were measured. Several study designs will be included, such as cohort studies, case–control studies, randomized controlled trials (RCTs), or systematic reviews on the topic. Qualitative studies, case-reports, abstracts, conference material, and editorials or commentaries will be excluded because these sources of information are unlikely to present the level of detail and information required for the purposes of this systematic review. Ecological studies will be excluded because they will not provide sufficient evidence for individual-level effects of comorbidity on rehabilitation outcome in patients with nTBI. Finally, although stroke may be considered an etiology of nTBI, it will be excluded from this review to reflect current research and clinical practice in rehabilitation—for example, various national rehabilitation information systems [20, 21] and rehabilitation centers [22, 23] classify stroke separately from nTBI. Further, a definition of nTBI that includes some vascular conditions, such as aneurysms, malformations, and subarachnoid hemorrhage, while excluding stroke, has been employed previously (e.g., [2, 3, 24–26]). Therefore, these vascular insults will be included.

The presentation of results will be guided by the Preferred Reporting Items for Systematic Reviews and Meta-Analyses (PRISMA) checklist [27].

### Data extraction

The standard data extraction table will include authors, publication year, purpose of study, year of study, study design, geographical location of study, setting (single center, multi-center, or population-based), data source or type (administrative, medical records, etc.), type and definition of nTBI population, statistical methods (e.g., descriptive, analytic, or stratification techniques), age of population, comorbidity measurement method (e.g., single comorbidity, count, type, or index), rationale, justification, or validation of the comorbidity measurement, outcome variables, and results involving comorbidity (see Additional file 2 for an example of the data extraction template).

To address the primary research objective, the measurement method (e.g., CCI, Elixhauser, etc.) will be listed in the column titled ‘comorbidity measurement’ (see Additional file 2). This information will provide a starting point to identify comorbidity measures that can be compared in future validation studies, should a validation study be conducted. To address the secondary research objective, measures of predictive power or model fit statistics, such as c-statistics for logistic regression and *R*^2^ values for linear regression, will be extracted if the study used regression techniques and reported these statistics. Face validity will be assessed by the rationale or justification provided by the authors for using a particular comorbidity measurement. This information will be extracted in the column titled ‘comorbidity measure rationale/justification/validation.’ If two or more studies are found to compare the predictive validity of various comorbidity measures within a nTBI population, this will be summarized and assessed as described below. In addition, these results can be confirmed in future validation and predictive modeling studies. To address the tertiary objective, any result related to the comorbidity measurement method used will be extracted. For example, if the CCI is used, the CCI score will be recorded. This information will be useful to health-care professionals by providing a summary of the profile of comorbidities in patients with nTBI within inpatient rehabilitation settings.

### Quality assessment

Critical Appraisal Skills Programme (CASP) checklists [28] will be used to assess study quality by guiding the assessment of validity and reliability of included studies. The checklists can be applied to various study designs including systematic reviews, RCTs, cohort, and case–control studies, which will allow for the assessment of the range of study designs that may be included in this systematic review. Methodological quality, such as the use of appropriate statistical tests, accounting for confounding, and the sample or population selection, is of particular importance as it can affect the interpretations and generalizability of results. The validity of comorbidity measurements used in the identified studies will be examined by (1) the face validity based on the authors’ rationale for using the measurement method, and (2) whether the predictive validity of the measurement method identified has been compared to other comorbidity measurement methods for the outcome in question for patients with nTBI in inpatient rehabilitation settings.

### Analyses

The analyses will depend on the search results and included studies. Due to the infancy of this field of research and thus the expected lack of validation studies, a narrative synthesis, similar to Buck et al. [16], will be conducted and guided by the Guidance for Narrative Synthesis in Systematic Reviews [29]. Specifically, the techniques of ‘tabulation,’ ‘grouping and clusters,’ and ‘textual description’ will be used. Extracted data will be tabulated as defined in the data extraction section and then grouped into clusters based on empirically important variables, such as the type of data used (e.g., administrative health data, chart reviews, or self-report), population stratification (e.g., nTBI vs. TBI, or anoxia vs. tumor vs. infection, etc.), and type of comorbidity measurement (number and type from ICD code, CCI, Elixhauser, etc.). Narrative synthesis will entail ‘textual descriptions,’ or the use of words and text to summarize and explain the methodology of comorbidity measurement for this population based on the grouping variables. Information on the type of comorbidity measurements will be summarized within the source of data since some measures have been developed for administrative data whereas others have not. In addition, information on the type of comorbidity measurement method used will be summarized within geographical region because health service research techniques are regionally specific. Narrative synthesis of the results of the included studies (i.e., the description or association of comorbidity and rehabilitation outcome) will be stratified by population and type of comorbidity measurement method used because comorbidities differ by population and how they are measured. The quality of the studies will also be described in the narrative synthesis.

In addition, meta-analyses will be conducted if appropriate. Since the comparison of the predictive validity of comorbidity measurement methods for risk adjustment is an emerging field, there has yet to be an established gold standard measurement for comorbidity in order to predict a given outcome. Also, it is unlikely that there will be a gold standard for risk adjustment, generally, due to the diversity of variables considered and the idiosyncratic development and validation methods used. However, if at least two studies are found to assess the predictive validity of at least two comorbidity measurement methods, a new meta-analytical technique will be applied, in which a hypergeometric test will be used to identify measurement methods with significantly superior or inferior performance for predicting functional outcome and length of stay [17]. Meta-analyses will be used to combine information on the comorbidity results to better understand the profile of comorbidity in patients with nTBI.

## Discussion

To the best of our knowledge, this is the first systematic review protocol to survey comorbidity measurement methods used in the nTBI rehabilitation population. Previous studies on the performance of comorbidity measurement methods have not focused on nTBI nor have they focused on inpatient rehabilitation [16], though this is considered to be important because extrapolation of comorbidity measurement and risk adjustment techniques from differing populations may not be valid [5, 13]. Moreover, the focus on nTBI, excluding stroke, is important as it facilitates investigation of a population that is not well studied as whole, particularly in the rehabilitation setting.

Due to the expected paucity of literature on this topic, the search terms were developed for high sensitivity using general terms, such as ‘brain injury,’ as well as high specificity using specific terms, such as ‘glioma.’ Proxy terms were developed and included for all three concepts. For example, the terms ‘cardiac arrest,’ ‘drowning,’ ‘choking,’ and ‘suffocation’ were included for the anoxic brain injury condition, as these events often result in this type of injury [30, 31]. Similarly, ‘brain irradiation’ was included to capture literature that may study brain damage due to cancer-related treatments [32]. For the comorbidity concept, the terms ‘epidemiologic factors,’ ‘age factors,’ and ‘sex factors’ were used to capture articles that may also measure comorbidity. Surrogate terms for comorbidity [16], common comorbidity indexes [33], and risk adjustment models (e.g., Diagnosis Related Groups) that incorporate comorbidity information were also included. Terms that are commonly used to capture literature related to rehabilitation outcomes, such as ‘length of stay’ or ‘functional independence,’ were used for the rehabilitation concept. As such, this protocol benefits from high sensitivity with the search terms. Due to the inherent imprecision of index terms, the use of general terms is expected to increase the number results from irrelevant populations, such as stroke or traumatic brain injury. However, with an expected paucity of literature, high sensitivity is desirable as it increases the likelihood of capturing relevant publications. Moreover, the specific definition of comorbidity used in this protocol addresses issues of conceptual ambiguity that have been previously identified [34].

However, these strengths have associated limitations. The increased sensitivity of this search strategy is expected to increase the number of irrelevant records, which requires more time for the screening process. Moreover, since the definition of comorbidity used to develop the search terms is limited to medical conditions or diagnoses, it does not include symptoms, function, or procedures, which are sometimes defined as comorbidity and may affect outcomes [16]. Finally, stroke has been excluded in this review to reflect research and clinical practice. Nonetheless, it may be considered a form of nTBI, and thus, future efforts should be directed at a separate review specifically for patients with stroke in rehabilitation settings in order to understand the impact of comorbidities in a manner that reflects the structure of research and clinical rehabilitation settings. Although a recently published systematic review on comorbidity measurement in cardiovascular disease research by Buck et al. [16] included a stroke population, it did not capture studies on stroke rehabilitation. At least two studies exist which show that comorbidity negatively affects functional outcome after stroke, and the method of comorbidity measurement affects the predictive validity of various rehabilitation outcomes [35, 36]. Thus, a future review on comorbidity measurement methods in patients with stroke should be conducted with a focus on rehabilitation outcomes.

This systematic review protocol allows for a comprehensive review of comorbidity measurement in the nTBI population in inpatient rehabilitation. This will provide insights into the current state of the literature, which can be used to guide future research on comorbidity measurement and risk adjustment for rehabilitation outcomes for this population. The results of the review may also provide a synthesis of the profile of comorbidity in patients with nTBI in inpatient rehabilitation and their rehabilitation outcomes. Most importantly, identification of the most appropriate techniques for comorbidity measurement for this population informs best practices for epidemiologic or health service research. This allows for a more accurate prediction of various rehabilitation outcomes for patients with nTBI with comorbidities, which will contribute to enhanced planning and delivery rehabilitation services.

## Electronic supplementary material

Additional file 1: **Database specific search terms.** Additional file 1 contains the search terms used in MEDLINE, Embase, The Cochrane Database of Systematic Reviews, PsycINFO, and the Health and Psychosocial Instruments (HAPI) databases. (DOCX 38 KB)

Additional file 2: **Example of data extraction table.** Additional file 2 contains an example of data extraction table that will be used to tabulate and group the information retrieved from the systematic review. (XLSX 12 KB)

## Abbreviations

ADGs: Aggregated Diagnosis Groups
CASP: Critical Appraisal Skills Programme
CCI: Charlson Comorbidity Index
CMS: Centers for Medicare and Medicaid Services
nTBI: Non-traumatic brain injury
PRISMA: Preferred Reporting Items for Systematic Reviews and Meta-Analyses
RCT: Randomized controlled trial.
