# Supplementary material for: A systematic review protocol for measuring comorbidity in inpatient rehabilitation for non-traumatic brain injury
Source: Syst Rev. 2015 Jan 29;4(1):14. doi: 10.1186/2046-4053-4-14 (PMC4328947; doi:10.1186/2046-4053-4-14)
Supplement: Supplementary file 1 — Additional file 1: Database specific search terms. Additional file 1 contains the search terms used in MEDLINE, Embase, The Cochrane Database of Systematic Reviews, PsycINFO, and the Health and Psychosocial Instruments (HAPI) databases. (DOCX 38 KB) [file 13643_2014_330_MOESM1_ESM.docx]

**Appendix I**

**MEDLINE**

1. nervous system diseases/ or central nervous system diseases/ or brain diseases/ or brain injuries/ or brain injury, chronic/ or brain damage, chronic/ or hypothalamic diseases/ or thalamic diseases/ or cerebellar diseases/ or encephalomalacia/

2. (((brain or neuro* or cereb* or crani* or CNS or central nervous system or intracranial or intracerebral or extradural or subdural or arachnoid or colloid or hypothalam* or thalam* or pituitary or pineal or primitive neuroectodermal or tentori* or supratentori* or infratentori* or choroid plexus or epidural or mening* or perimening*) adj3 (disease* or injur* or insult* or d?sfunction* or disorder* or damage* or lesion*)) or encephalopath* or encephalomalacia or cerebral softening).tw.

3. ((nontraumatic or non traumatic or acquired) adj3 (brain or neuro* or cereb* or CNS or central nervous system) adj3 (disease* or injur* or insult* or d?sfunction* or disorder* or damage* or lesion*)).tw.

4. or/1-3

5. exp brain neoplasms/ or exp central nervous system cysts/ or exp meningeal neoplasms/

6. (((brain or neuro* or cereb* or crani* or CNS or central nervous system or intracranial or intracerebral or extradural or subdural or arachnoid or colloid or hypothalam* or thalam* or pituitary or pineal or primitive neuroectodermal or tentori* or supratentori* or infratentori* or choroid plexus or epidural or mening* or perimening*) adj3 (neoplasm* or lesion* or tumo?r* or cyst* or granuloma* or lymphoma* or germ cell tumo?r* or cancer* or adenoma* or macroadenoma*)) or (neurooncology or neuro oncology)).tw.

7. (glioma* or astrocytoma* or oligodendroglioma* or ependymoma* or glioblastoma multiforme* or meningioma* or haemangioblastoma* or acoustic neuroma* or vestibular schwannomas* or craniopharyngioma* or medulloblastoma* or haemangiopericytoma* or neurocytoma* or schwannoma* or chordoma* or pinealoma* or leptomeningeal carcinomatosis or choroid plexus papilloma*).tw.

8. (brain adj3 (irradiation or radiation)).tw.

9. or/5-8

10. exp central nervous system infections/ or exp encephalomyelitis/

11. ((brain or neuro* or cereb* or crani* or CNS or central nervous system or intracranial or intracerebral or extradural or subdural or arachnoid or colloid or hypothalam* or thalam* or pituitary or pineal or primitive neuroectodermal or tentori* or supratentori* or infratentori* or choroid plexus or epidural or mening* or perimening*) adj3 (infection* or inflammation* or abscess* or toxoplasmosis or empyema* or ventriculit* or effusion or tuberculom* or malaria or tuberculosis or helminthiasis or vasculitis)).tw.

12. (encephalit* or meningit* or encephalomyelitis or encephalomeningit* or meningoencephalit* or arachnoiditi* or leukoencepha* or panencephaliti* or choriomengiti* or polioencephal* or neuroborreliosis or neurosyphili* or neuroaspergillosis* or neurocysticercosis or neuroschistosomiasis).tw.

13. or/10-12

14. exp hypoxia, brain/ or asphyxia/ or exp heart arrest/ or exp drowning/ or anoxia/

15. (anoxi* or hypoxi* or asphyxia* or drown* or 'cardiac arrest' or 'heart arrest' or strangl* or 'carbon monoxide' or chok* or suffocat*).tw.

16. or/14-15

17. exp neurotoxicity syndromes/

18. ((neurotoxic* or 'toxic brain' or 'organic brain') adj3 (syndrom* or injur* or disease*)).tw.

19. ((brain or cereb* or neuro* or enceph* or CNS or central nervous system) adj3 (poison* or toxic* or intoxication) adj3 (cocaine or heroin or antiepileptic or sedative or hypnotic or methaqualone or valproic acid or alcohol or metal or lead or arsenic or manganese or hydrogen cyanide or carbon monoxide or cyanide or aflatoxin or mycotoxin)).tw.

20. (drug induced akathisia or alcohol induced nervous system disorder* or botulism or drug induced dyskinesia or heavy metal nervous system poisoning or MPTP poisoning or neuroleptic malignant syndrome).tw.

21. or/17-20

22. exp intracranial hypertension/ or pneumocephalus/ or brain edema/ or meningism/ or high pressure neurological syndrome/ or exp hydrocephalus/ or intracranial hypotension/

23. (((brain or neuro* or cereb* or intracranial or intracerebral) adj3 (oedema or edema or compress*)) or hydrocephalus or intracranial hypertens* or meningism or meningismus or pseudotumor cerebri or pneumocephalus or intracranial hypotens*).tw.

24. or/22-23

25. exp brain diseases, metabolic/

26. ((metabol* or diabet* or hypoglyc?em*) adj3 (brain disease* or brain injur* or neuro* disease* or neuro* injur* or coma)).tw.

27. (kernicterus or hepatic encephalopath* or marchiafava bignami* or central pontine myelinosis or reye syndrome or wernicke encephalopathy).tw.

28. or/25-27

29. exp hematoma, subdural/ or intracranial hemorrhages/ or intracranial aneurysm/ or exp central nervous system vascular malformations/

30. (((subdural or intracranial) adj3 h?emorrhage) or brain aneur?sm or cerebral aneur?sm or intracranial aneur?sm or arteriovenous malformation or 'AVM').tw.

31. or/29-30

32. 4 or 9 or 13 or 16 or 21 or 24 or 28 or 31

33. exp Comorbidity/

34. exp Diagnosis-Related Groups/

35. exp Risk Adjustment/

36. Epidemiologic Factors/ or Risk Factors/ or Age Factors/ or Sex Factors/

37. (comorbid* or co morbid* or multimorbid* or multi morbid*).tw.

38. ((clinical* or medical*) adj3 (characteristics* or complex* or histor*)).tw.

39. ((coexist* or co exist* or cooccur* or co occur*) adj3 (illness* or disease* or condition* or complication* or diagnos* or risk*)).tw.

40. ('charlson comorbidity index' or 'CCI' or 'CMI' or elixhauser or 'BOD index' or 'cumulative index rating scale' or 'CIRS' or 'Coroni-Huntley index' or 'DUSOI index' or 'Hallstrom index' or 'Hurwitz index' or 'Incalzi index', 'Kaplan index', 'Liu index', 'Shwartz index').tw.

41. ('diagnosis related group*' or 'DRG' or 'case mix' or 'casemix' or 'risk adjust*' or resource intensity weight* or RWI).tw.

42. ((epidemiologic or risk or age or sex or gender or predisposing or enabling or need*) adj3 (factor* or variable*)).tw.

43. (andersen model or andersen behavio?ral model).tw.

44. or/33-43

45. exp "Physical and Rehabilitation Medicine"/ or exp Rehabilitation Nursing/ or exp Rehabilitation Centers/ or exp Rehabilitation/ or exp Rehabilitation, Vocational/ or exp Length of Stay/ or exp Recovery of Function/

46. rehabilitation.fs.

47. 'rehabilitat*'.tw.

48. ('length of stay' or 'LOS').tw.

49. ('functional outcome' or 'functional independence' or 'functional independence measure' or 'FIM' or 'recovery of function' or 'functional recovery' or 'discharge destination' or 'discharge status').tw.

50. or/45-49

51. 32 and 44 and 50

52. animals/ not (animals/ and humans/)

53. 51 not 52

54. limit 53 to english language

**EMBASE**

1. neurologic disease/ or central nervous system disease/ or brain disease/ or brain injury/ or acquired brain injury/ or brain damage/ or brain stem injury/ or cerebellum injury/ or chronic brain disease/ or acute brain disease/ or brain dysfunction/ or cerebellum disease/ or encephalomalacia/

2. (((brain or neuro* or cereb* or crani* or CNS or central nervous system or intracranial or intracerebral or extradural or subdural or arachnoid or colloid or hypothalam* or thalam* or pituitary or pineal or primitive neuroectodermal or tentori* or supratentori* or infratentori* or choroid plexus or epidural or mening* or perimening*) adj3 (disease* or injur* or insult* or d?sfunction* or disorder* or damage* or lesion*)) or encephalopath* or encephalomalacia or cerebral softening).tw.

3. ((nontraumatic or non traumatic or acquired) adj3 (brain or neuro* or cereb* or CNS or central nervous system) adj3 (disease* or injur* or insult* or d?sfunction* or disorder* or damage* or lesion*)).tw.

4. or/1-3

5. exp central nervous system tumor/ or brain cyst/ or colloid cyst/ or brain abscess/ or brain radiation/ or arachnoid cyst/

6. (((brain or neuro* or cereb* or crani* or CNS or central nervous system or intracranial or intracerebral or extradural or subdural or arachnoid or colloid or hypothalam* or thalam* or pituitary or pineal or primitive neuroectodermal or tentori* or supratentori* or infratentori* or choroid plexus or epidural or mening* or perimening*) adj3 (neoplasm* or lesion* or tumo?r* or cyst* or granuloma* or lymphoma* or germ cell tumo?r* or cancer* or adenoma* or macroadenoma*)) or (neurooncology or neuro oncology)).tw.

7. (glioma* or astrocytoma* or oligodendroglioma* or ependymoma* or glioblastoma multiforme* or meningioma* or haemangioblastoma* or acoustic neuroma* or vestibular schwannomas* or craniopharyngioma* or medulloblastoma* or haemangiopericytoma* or neurocytoma* or schwannoma* or chordoma* or pinealoma* or leptomeningeal carcinomatosis or choroid plexus papilloma*).tw.

8. (brain adj3 (irradiation or radiation)).tw.

9. or/5-8

10. exp central nervous system infection/ or encephalomyelitis/

11. (encephalit* or meningit* or encephalomyelitis or encephalomeningit* or meningoencephalit* or arachnoiditi* or leukoencepha* or panencephaliti* or choriomengiti* or polioencephal* or neuroborreliosis or neurosyphili* or neuroaspergillosis* or neurocysticercosis or neuroschistosomiasis).tw.

12. ((brain or neuro* or cereb* or crani* or CNS or central nervous system or intracranial or intracerebral or extradural or subdural or arachnoid or colloid or hypothalam* or thalam* or pituitary or pineal or primitive neuroectodermal or tentori* or supratentori* or infratentori* or choroid plexus or epidural or mening* or perimening*) adj3 (infection* or inflammation* or abscess* or toxoplasmosis or empyema* or ventriculit* or effusion or tuberculom* or malaria or tuberculosis or helminthiasis or vasculitis)).tw.

13. or/10-12

14. brain hypoxia/ or hypoxic ischemic encephalopathy/ or asphyxia/ or suffocation/ or strangulation/ or exp heart arrest/ or exp drowning/ or airway obstruction/ or anoxia/

15. (anoxi* or hypoxi* or asphyxia* or drown* or 'cardiac arrest' or 'heart arrest' or strangl* or 'carbon monoxide' or chok* or suffocat* or airway obstruction).tw.

16. or/14-15

17. neurotoxicity/ or delayed neurotoxicity/ or brain toxicity/ or organic brain syndrome/ or organic psychosyndrome/ or toxicity/ or exp intoxication/ or drug toxicity/ or akathisia/ or dyskinesia/ or neuroleptic malignant syndrome/

18. ((neurotoxic* or 'toxic brain' or 'organic brain') adj3 (syndrom* or injur* or disease*)).tw.

19. ((brain or cereb* or neuro* or enceph* or CNS or central nervous system) adj3 (poison* or toxic* or intoxication) adj3 (cocaine or heroin or antiepileptic or sedative or hypnotic or methaqualone or valproic acid or alcohol or metal or lead or arsenic or manganese or hydrogen cyanide or carbon monoxide or cyanide or aflatoxin or mycotoxin)).tw.

20. (drug induced akathisia or alcohol induced nervous system disorder* or botulism or drug induced dyskinesia or heavy metal nervous system poisoning or MPTP poisoning or neuroleptic malignant syndrome).tw.

21. or/17-20

22. exp intracranial hyperstension/ or brain edema/ or hydrocephalus/ or brain aqueduct stenosis/ or brain ventricle dilation/ or communicating hydrocephalus/ or normotensive hydrocephalus/ or obstructive hydrocephalus/ or pneumocephalus/ or hypertension encephalopathy/ or brain pseudotumor/ or meningism/ or intracranial hypotension/ or high pressure neurological syndrome/

23. (((brain or neuro* or cereb* or intracranial or intracerebral) adj3 (oedema or edema or compress*)) or hydrocephalus or intracranial hypertens* or meningism or meningismus or pseudotumor cerebri or intracranial hypotens*).tw.

24. or/22-23

25. exp metabolic encephalopathy/ or marchiafava bignami disease/

26. ((metabol* or diabet* or hypoglyc?em*) adj3 (brain disease* or brain injur* or neuro* disease* or neuro* injur* or coma)).tw.

27. (kernicterus or hepatic encephalopath* or marchiafava bignami* or central pontine myelinosis or reye syndrome or wernicke encephalopathy).tw.

28. or/25-27

29. subdural hematoma/ or brain hemorrhage/ or cerebrovascular malformation/ or exp intracranial anerysm/ or brain arteriovenous malformation/ or central nervous system malformation/

30. (((subdural or intracranial) adj3 h?emorrhage) or brain aneur?sm or cerebral aneur?sm or intracranial aneur?sm or arteriovenous malformation or 'AVM').tw.

31. or/29-30

32. 4 or 9 or 13 or 16 or 21 or 24 or 28 or 31

33. comorbidity/ or Charlson Comorbidity Index/ or Elixhauser Comorbidity Index/ or case mix/ or diagnosis related group/ or risk assessment/ or epidemiologic data/ or age/ or sex difference/

34. (comorbid* or co morbid* or multimorbid* or multi morbid*).tw.

35. ((clinical* or medical*) adj3 (characteristics* or complex* or histor*)).tw.

36. ((coexist* or co exist* or cooccur* or co occur*) adj3 (illness* or disease* or condition* or complication* or diagnos* or risk*)).tw.

37. ('charlson comorbidity index' or 'CCI' or 'CMI' or elixhauser or 'BOD index' or 'cumulative index rating scale' or 'CIRS' or 'Coroni-Huntley index' or 'DUSOI index' or 'Hallstrom index' or 'Hurwitz index' or 'Incalzi index', 'Kaplan index', 'Liu index', 'Shwartz index').tw.

38. ('diagnosis related group*' or 'DRG' or 'case mix' or 'casemix' or 'risk adjust*' or resource intensity weight* or RWI).tw.

39. ((epidemiologic or risk or age or sex or gender or predisposing or enabling or need*) adj3 (factor* or variable*)).tw.

40. (Andersen model or Andersen behavio?ral model).tw.

41. or/33-40

42. rehabilitation center/ or rehabilitation research/ or rehabilitation patient/ or speech rehabilitation/ or exp rehabilitation/ or rehabilitation medicine/ or rehabilitation nursing/ or psychosocial rehabilitation/ or vocational rehabilitation/ or cognitive rehabilitation/ or rehabilitation care/ or length of stay/ or convalescence/

43. rh.fs.

44. 'rehabilitat*'.tw.

45. ('length of stay' or 'LOS').tw.

46. ('functional outcome' or 'functional independence' or 'functional independence measure' or 'FIM' or 'recovery of function' or 'functional recovery' or 'discharge destination' or 'discharge status').tw.

47. or/42-46

48. 32 and 41 and 47

49. limit 48 to English language

**PsycINFO**

1. nervous system disorders/ or central nervous system disorders/ or brain disorders/ or brain damage/ or encephalopathies/

2. (((brain or neuro* or cereb* or crani* or CNS or central nervous system or intracranial or intracerebral or extradural or subdural or arachnoid or colloid or hypothalam* or thalam* or pituitary or pineal or primitive neuroectodermal or tentori* or supratentori* or infratentori* or choroid plexus or epidural or mening* or perimening*) adj3 (disease* or injur* or insult* or d?sfunction* or disorder* or damage* or lesion*)) or encephalopath* or encephalomalacia or cerebral softening).tw.

3. ((nontraumatic or non traumatic or acquired) adj3 (brain or neuro* or cereb* or CNS or central nervous system) adj3 (disease* or injur* or insult* or d?sfunction* or disorder* or damage* or lesion*)).tw.

4. (anoxi* or hypoxi* or asphyxia* or drown* or 'cardiac arrest' or 'heart arrest' or strangl* or 'carbon monoxide' or chok* or suffocat*).tw.

5. brain neoplasms/ or glioma/ or intracranial abcesses/

6. (((brain or neuro* or cereb* or crani* or CNS or central nervous system or intracranial or intracerebral or extradural or subdural or arachnoid or colloid or hypothalam* or thalam* or pituitary or pineal or primitive neuroectodermal or tentori* or supratentori* or infratentori* or choroid plexus or epidural or mening* or perimening*) adj3 (neoplasm* or lesion* or tumo?r* or cyst* or granuloma* or lymphoma* or germ cell tumo?r* or cancer* or adenoma* or macroadenoma*)) or (neurooncology or neuro oncology)).tw.

7. (glioma* or astrocytoma* or oligodendroglioma* or ependymoma* or glioblastoma multiforme* or meningioma* or haemangioblastoma* or acoustic neuroma* or vestibular schwannomas* or craniopharyngioma* or medulloblastoma* or haemangiopericytoma* or neurocytoma* or schwannoma* or chordoma* or pinealoma* or leptomeningeal carcinomatosis or choroid plexus papilloma*).tw.

8. (brain adj3 (irradiation or radiation)).tw.

9. exp encephalitis/ or exp meningitis/ or neurosyphilis/

10. ((brain or neuro* or cereb* or crani* or CNS or central nervous system or intracranial or intracerebral or extradural or subdural or arachnoid or colloid or hypothalam* or thalam* or pituitary or pineal or primitive neuroectodermal or tentori* or supratentori* or infratentori* or choroid plexus or epidural or mening* or perimening*) adj3 (infection* or inflammation* or abscess* or toxoplasmosis or empyema* or ventriculit* or effusion or tuberculom* or malaria or tuberculosis or helminthiasis or vasculitis)).tw.

11. (encephalit* or meningit* or encephalomyelitis or encephalomeningit* or meningoencephalit* or arachnoiditi* or leukoencepha* or panencephaliti* or choriomengiti* or polioencephal* or neuroborreliosis or neurosyphili* or neuroaspergillosis* or neurocysticercosis or neuroschistosomiasis).tw.

12. ((metabol* or diabet* or hypoglyc?em*) adj3 (brain disease* or brain injur* or neuro* disease* or neuro* injur* or coma)).tw.

13. (kernicterus or hepatic encephalopath* or marchiafava bignami* or central pontine myelinosis or reye syndrome or wernicke encephalopathy).tw.

14. toxic encephalopathies/ or wernicke's syndrome/ or acute alcoholic intoxication/ or chronic alcoholic intoxication/ or organic brain syndromes/ or alcoholic psychosis/ or toxic psychosis/

15. ((neurotoxic* or 'toxic brain' or 'organic brain') adj3 (syndrom* or injur* or disease*)).tw.

16. ((brain or cereb* or neuro* or enceph* or CNS or central nervous system) adj3 (poison* or toxic* or intoxication) adj3 (cocaine or heroin or antiepileptic or sedative or hypnotic or methaqualone or valproic acid or alcohol or metal or lead or arsenic or manganese or hydrogen cyanide or carbon monoxide or cyanide or aflatoxin or mycotoxin)).tw.

17. (((subdural or intracranial) adj3 h?emorrhage) or brain aneur?sm or cerebral aneur?sm or intracranial aneur?sm or arteriovenous malformation or 'AVM').tw.

18. hydrocephalus/

19. (((brain or neuro* or cereb* or intracranial or intracerebral) and (oedema or edema or compress*)) or hydrocephalus or intracranial hypertens* or meningism or meningismus or pseudotumor cerebri or pneumocephalus or intracranial hypotens*).tw.

20. or/1-19

21. comorbidity/ or risk factors/

22. (comorbid* or co morbid* or multimorbid* or multi morbid*).tw.

23. ((clinical* or medical*) adj3 (characteristics* or complex* or histor*)).tw.

24. ((coexist* or co exist* or cooccur* or co occur*) adj3 (illness* or disease* or condition* or complication* or diagnos* or risk*)).tw.

25. ('charlson comorbidity index' or 'CCI' or 'CMI' or elixhauser or 'BOD index' or 'cumulative index rating scale' or 'CIRS' or 'Coroni-Huntley index' or 'DUSOI index' or 'Hallstrom index' or 'Hurwitz index' or 'Incalzi index', 'Kaplan index', 'Liu index', 'Shwartz index').tw.

26. ('diagnosis related group*' or 'DRG' or 'case mix' or 'casemix' or 'risk adjust*' or resource intensity weight* or RWI).tw.

27. ((epidemiologic or risk or age or sex or gender or predisposing or enabling or need*) adj3 (factor* or variable*)).tw.

28. (Andersen model or Andersen behavio?ral model).tw.

29. or/21-28

30. exp neuropsychological rehabilitation/ or exp psychosocial rehabilitation/ or rehabilitation/ or rehabilitation centers/ or rehabilitation counseling/ or vocational rehabilitation/ or neurorehabilitation/ or occupational therapy/ or physical therapy/ or treatment duration/ or "recovery (disorders)"/

31. 'rehabilitat*'.tw.

32. ('length of stay' or 'LOS').tw.

33. ('functional outcome' or 'functional independence' or 'functional independence measure' or 'FIM' or 'recovery of function' or 'functional recovery' or 'discharge destination' or 'discharge status').tw.

34. or/30-33

35. 20 and 29 and 34

36. limit 35 to English language

**CDSR and HAPI**

1. (((brain or neuro* or cereb* or crani* or CNS or central nervous system or intracranial or intracerebral or extradural or subdural or arachnoid or colloid or hypothalam* or thalam* or pituitary or pineal or primitive neuroectodermal or tentori* or supratentori* or infratentori* or choroid plexus or epidural or mening* or perimening*) adj3 (disease* or injur* or insult* or d?sfunction* or disorder* or damage* or lesion*)) or encephalopath* or encephalomalacia or cerebral softening).tw.

2. ((nontraumatic or non traumatic or acquired) adj3 (brain or neuro* or cereb* or CNS or central nervous system) adj3 (disease* or injur* or insult* or d?sfunction* or disorder* or damage* or lesion*)).tw.

3. (anoxi* or hypoxi* or asphyxia* or drown* or 'cardiac arrest' or 'heart arrest' or strangl* or 'carbon monoxide' or chok* or suffocat*).tw.

4. (((brain or neuro* or cereb* or crani* or CNS or central nervous system or intracranial or intracerebral or extradural or subdural or arachnoid or colloid or hypothalam* or thalam* or pituitary or pineal or primitive neuroectodermal or tentori* or supratentori* or infratentori* or choroid plexus or epidural or mening* or perimening*) adj3 (neoplasm* or lesion* or tumo?r* or cyst* or granuloma* or lymphoma* or germ cell tumo?r* or cancer* or adenoma* or macroadenoma*)) or (neurooncology or neuro oncology)).tw.

5. (glioma* or astrocytoma* or oligodendroglioma* or ependymoma* or glioblastoma multiforme* or meningioma* or haemangioblastoma* or acoustic neuroma* or vestibular schwannomas* or craniopharyngioma* or medulloblastoma* or haemangiopericytoma* or neurocytoma* or schwannoma* or chordoma* or pinealoma* or leptomeningeal carcinomatosis or choroid plexus papilloma*).tw.

6. (brain adj3 (irradiation or radiation)).tw.

7. ((brain or neuro* or cereb* or crani* or CNS or central nervous system or intracranial or intracerebral or extradural or subdural or arachnoid or colloid or hypothalam* or thalam* or pituitary or pineal or primitive neuroectodermal or tentori* or supratentori* or infratentori* or choroid plexus or epidural or mening* or perimening*) adj3 (infection* or inflammation* or abscess* or toxoplasmosis or empyema* or ventriculit* or effusion or tuberculom* or malaria or tuberculosis or helminthiasis or vasculitis)).tw.

8. (encephalit* or meningit* or encephalomyelitis or encephalomeningit* or meningoencephalit* or arachnoiditi* or leukoencepha* or panencephaliti* or choriomengiti* or polioencephal* or neuroborreliosis or neurosyphili* or neuroaspergillosis* or neurocysticercosis or neuroschistosomiasis).tw.

9. ((metabol* or diabet* or hypoglyc?em*) adj3 (brain disease* or brain injur* or neuro* disease* or neuro* injur* or coma)).tw.

10. (kernicterus or hepatic encephalopath* or marchiafava bignami* or central pontine myelinosis or reye syndrome or wernicke encephalopathy).tw.

11. ((neurotoxic* or 'toxic brain' or 'organic brain') adj3 (syndrom* or injur* or disease*)).tw.

12. ((brain or cereb* or neuro* or enceph* or CNS or central nervous system) adj3 (poison* or toxic* or intoxication) adj3 (cocaine or heroin or antiepileptic or sedative or hypnotic or methaqualone or valproic acid or alcohol or metal or lead or arsenic or manganese or hydrogen cyanide or carbon monoxide or cyanide or aflatoxin or mycotoxin)).tw.

13. (((subdural or intracranial) adj3 h?emorrhage) or brain aneur?sm or cerebral aneur?sm or intracranial aneur?sm or arteriovenous malformation or 'AVM').tw.

14. (((brain or neuro* or cereb* or intracranial or intracerebral) and (oedema or edema or compress*)) or hydrocephalus or intracranial hypertens* or meningism or meningismus or pseudotumor cerebri or pneumocephalus or intracranial hypotens*).tw.

15. or/1-14

16. (comorbid* or co morbid* or multimorbid* or multi morbid*).tw.

17. ((clinical* or medical*) adj3 (characteristics* or complex* or histor*)).tw.

18. ((coexist* or co exist* or cooccur* or co occur*) adj3 (illness* or disease* or condition* or complication* or diagnos* or risk*)).tw.

19. ('charlson comorbidity index' or 'CCI' or 'CMI' or elixhauser or 'BOD index' or 'cumulative index rating scale' or 'CIRS' or 'Coroni-Huntley index' or 'DUSOI index' or 'Hallstrom index' or 'Hurwitz index' or 'Incalzi index', 'Kaplan index', 'Liu index', 'Shwartz index').tw.

20. ('diagnosis related group*' or 'DRG' or 'case mix' or 'casemix' or 'risk adjust*' or resource intensity weight* or RWI).tw.

21. ((epidemiologic or risk or age or sex or gender or predisposing or enabling or need*) adj3 (factor* or variable*)).tw.

22. (Andersen model or Andersen behavio?ral model).tw.

23. or/16-22

24. 'rehabilitat*'.tw.

25. ('length of stay' or 'LOS').tw.

26. ('functional outcome' or 'functional independence' or 'functional independence measure' or 'FIM' or 'recovery of function' or 'functional recovery' or 'discharge destination' or 'discharge status').tw.

27. or/24-26

28. 15 and 23 and 27

29. limit 28 to English language
